# Ice ingestion with a long rest interval increases the endurance exercise capacity and reduces the core temperature in the heat

**DOI:** 10.1186/s40101-016-0122-6

**Published:** 2017-01-05

**Authors:** Takashi Naito, Yuka Iribe, Tetsuro Ogaki

**Affiliations:** Graduate School of Human-Environment Studies, Kyushu University, 6-1 Kasuga-koen, Kasuga-City, Fukuoka 816-8580 Japan

**Keywords:** Crushed ice, Internal cooling, Time to exhaustion, Rectal temperature

## Abstract

**Background:**

The timing in which ice before exercise should be ingested plays an important role in optimizing its success. However, the effects of differences in the timing of ice ingestion before exercise on cycling capacity, and thermoregulation has not been studied. The aim of the present study was to assess the effect of length of time after ice ingestion on endurance exercise capacity in the heat.

**Methods:**

Seven males ingested 1.25 g kg body mass^−1^ of ice (0.5 °C) or cold water (4 °C) every 5 min, six times. Under three separate conditions after ice or water ingestion ([1] taking 20 min rest after ice ingestion, [2] taking 5 min rest after ice ingestion, and [3] taking 5 min rest after cold water ingestion), seven physically active male cyclists exercised at 65% of their maximal oxygen uptake to exhaustion in the heat (35 °C, 30% relative humidity).

**Results:**

Participants cycled significantly longer following both ice ingestion with a long rest interval (46.0 ± 7.7 min) and that with a short rest interval (38.7 ± 5.7 min) than cold water ingestion (32.3 ± 3.2 min; both *p* < 0.05), and the time to exhaustion was 16% (*p* < 0.05) longer for ice ingestion with a long rest interval than that with a short rest interval. Ice ingestion with a long rest interval (−0.55 ± 0.07 °C; both *p* < 0.05) allowed for a greater drop in the core temperature than both ice ingestion with a short rest interval (−0.36 ± 0.16 °C) and cold water ingestion (−0.11 ± 0.14 °C). Heat storage under condition of ice ingestion with a long rest interval during the pre-exercise period was significantly lower than that observed with a short rest interval (−4.98 ± 2.50 W m^−2^; *p* < 0.05) and cold water ingestion (2.86 ± 4.44 W m^−2^).

**Conclusions:**

Therefore, internal pre-cooling by ice ingestion with a long rest interval had the greatest benefit on exercise capacity in the heat, which is suggested to be driven by a reduced rectal temperature and heat storage before the start of exercise.

## Background

A high core temperature is an independent cause of fatigue during exercise in a hot environment and results in a deterioration of exercise capacity [1–3]. In particular, the attainment of a critical core temperature has been suggested to be one of the main limiting factors inhibiting endurance exercise performance [4, 5]. Several strategies, such as pre-cooling, have been proposed to prevent hyperthermia and minimize exercise performance impairments in hot environments [6, 7].

The ingestion of ice, including crushed ice or ice slurry, has been recently suggested to be an effective and practical method of pre-cooling the core temperature and improving endurance exercise performance [8–11]. Indeed, ice ingestion has been demonstrated to lower the core temperature by 0.3–0.5 °C and improves endurance exercise performance by 0.6–19% [8–10]. Lowering the core temperature by ice ingestion allows for greater heat storage capacity during exercise, in turn delaying the onset of hyperthermia-induced fatigue [7]. Recently, however, some studies have found no beneficial effect of ice ingestion [12, 13]. These studies reported that participants began the endurance exercise within 5 min of consuming the final beverage, as such these strategies may not have been effective because of the short rest interval used.

The timing in which ice is ingested may be important for optimizing its effects. In most previous studies, subjects ingested ice over a 30-min period at a standardized rate that ranged from 5 min. However, Onitsuka et al. [14] found that when participants reminded at rest, their rectal temperature (Tre) kept on decreasing for approximately 20 min after following the end of 7.5 g kg body mass (BM)^−1^ ice slurry ingestion. Similarly, Naito and Ogaki [15] reported that intermittent ice ingestion at 1.25 g kgBM^−1^ every 5 min for 30 min continued to decrease the Tre until approximately 20 min after the end of ingestion; in their study, the Tre was reduced by 0.56 ± 0.20 °C compared to ingestion as a single bolus. Previous studies have suggested that the greater the magnitude by which the core temperature is reduced, the greater the performance benefit [7]. Thus, we thought that intermittent ice ingestion would be an even more effective strategy if subsequent exercise was started after a lower Tre had been achieved. It is possible that a novel pre-cooling strategy with a long rest interval (LRI; 20 min) after ice ingestion may reduce the pre-exercise core temperature and improve the endurance cycling capacity compared with a traditional strategy involving only a short rest interval (SRI; 5 min).

The purpose of this study was to investigate the effects of different rest intervals after ingestion on endurance cycling capacity and core temperature in the heat. We hypothesized that ice ingestion would result in a lower Tre and longer endurance cycling capacity than cold water ingestion. We further hypothesized that ice ingestion with a LRI would reduce Tre before exercise and improve endurance exercise capacity to a greater extent than that with a SRI.

## Methods

### Participants

Seven non-heat acclimatized, physically active male recreational cyclists (age = 25 ± 2 years; height = 1.74 ± 0.04 m; BM = 70.7 ± 12.3 kg; maximal oxygen uptake ($\overset{.}{\mathrm{V}}$ O_2_max) = 48.8 ± 4.7 mL kg^−1^min^−1^) were recruited for this study. The experiments were approved by the Ethics Committee of Human-Environment Studies, Kyushu University, and all participants read and signed an informed consent form before the experiments began.

### Preliminary measurements

In order to determine the $\overset{.}{\mathrm{V}}$ O_2_max, on their first visit to the laboratory, each participant performed a progressive exercise test on a cycle ergometer (Ergomedic 828 E; Monark, Varberg, Sweden) at room temperature (25 °C and 50% relative humidity (RH)). Their height and BM were measured to the nearest 0.1 cm and 10 g, respectively (TBF-210; Tanita Co., Tokyo, Japan). The protocol consisted of progressive exercise beginning at 90 W for 3 min, followed by increments of 30 W every 3 min until volitional exhaustion [16]. The participants were asked to maintain a pedal cadence of 60 rev min^−1^ throughout the progressive exercise test. The test was considered to be valid if two of the following three criteria were met: (1) oxygen consumption reached a plateau, (2) heart rate (HR) remained within 10% of the predicted maximum (220–age), or (3) the respiratory exchange ratio was above 1.05 [16]. On the second visit, between 4 and 14 days later, the participants performed a familiarization trial involving cycling to exhaustion at an intensity of 65% $\overset{.}{\mathrm{V}}$ O_2_max in the same hot environment as the experimental trials [4, 17].

### Experimental trials

In a randomized and crossover design, all of the participants performed three trials each as follows: ingesting crushed ice with a long (20-min) rest interval before exercise (LRI), crushed ice with a short (5-min) rest interval before exercise (SRI) or cold water with short (5-min) rest interval before exercise (CON). During the 24-h period before the experimental trial, the participants were instructed to avoid strenuous exercise as well as the consumption of alcohol, caffeine, nonsteroidal anti-inflammatory drugs, and nutritional supplements. All participants completed a diary that was replicated prior to the second and third trials. Each participant arrived at the laboratory after having refrained from eating for 6 h and drinking any type of beverage for 2 h. They were instructed to drink 500 mL of plain water 2 h before all tests to help promote euhydration prior to the start of each trial. For each participant, the three trials were commenced at the same time to control for circadian variations in the core temperature and were separated by 4–7 days.

Upon arrival at the laboratory, the participants had urine samples collected and were weighed before they entered a climate-controlled room (35 °C and 30% RH). A rectal thermistor (ITP010-11; Nikkiso-Therm Co., Ltd., Tokyo, Japan) was inserted approximately 15 cm into the rectum. Three skin thermistors were affixed using hypoallergenic polyacrylate adhesive tape (ITP082-24; Nikkiso-Therm Co., Ltd., Tokyo, Japan) at the chest, forearm, and thigh. A HR monitor was then fixed to each participant’s chest before a 5-min rest period to gather baseline data.

**Fig. 1:**
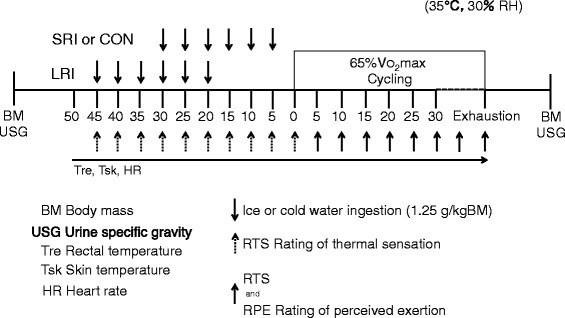
Schematic representation of the experimental protocol

Crushed ice was made using a commercially available food blender (TM8100; Tescom Co., Ltd., Tokyo, Japan). The participants were given 1.25 g kgBM^−1^ of ice (0.5 °C) or cold water (4 °C) every 5 min for 30 min. In the LRI trial, the participants commenced cycling exercise at an intensity equivalent to 65% $\overset{.}{\mathrm{V}}$ O_2_max until voluntary exhaustion, 20 min following the end of ice ingestion. In the SRI or CON trials, the participants mounted the cycle ergometer to start the cycling, 5 min after fully ingesting the last drink. The participants were asked to maintain a pedal cadence of 60 rev min^−1^ throughout the exercise. Exhaustion was defined as being unable to maintain 60 rev min^−1^ for 10 s. After the exercise period, the participants dried themselves with a towel and were weighed again to determine their BM before the collection of a final urine sample (Fig. 1).

### Measurements

The HR was monitored continuously throughout the trial using a HR monitor (DS-3140; Fukuda Denshi) and reported as the average for each 5-min interval. Urine samples were measured to evaluate hydration status by urine specific gravity (USG), which was determined using an analog USG scale (Atago, Japan). Throughout the three trials, the Tre and skin temperature were recorded continuously via a data logger (N542R; Nikkiso-Therm Co., Ltd.) and logged intermittently at 30-s intervals. The mean skin temperature (Tsk) was calculated using the formula from Roberts et al. [18]: Tsk = 0.43 × (Tchest) + 0.25 × (Tarm) + 0.32 × (Tthigh). The mean body temperature (Tb) was calculated using the formula from Colin et al. [19]: ΔTb = 0.8 × (ΔTre) + 0.2 × (ΔTsk) + 0.4.

Heat storage (HS) was calculated at 5-min increments using the formula described by Adams et al. [20]: HS = 0.965 × BM × ΔTb/AD, where 0.965 is the specific HS capacity of the body (W kg^−1^ °C^−1^) and AD is the body surface area (m^2^): AD = 0.202 × BM^0.425^ × height^0.725^ [21]. The total sweat loss (TSL) was calculated using the following formula: (BM before the experiment − BM after the experiment) + the amount of the ingested drink. A rating of the subjective thermal sensation [22] (RTS; 9-point scale ranging from 1 = “very cold” to 9 = “very hot”) was recorded every 5 min throughout each trial, while a rating of the perceived exertion (RPE; 15-point scale) was recorded every 5 min during exercise [23].

### Statistical analysis

All statistical computations were performed using the IBM SPSS Statistics 21 software package (SPSS, Inc., Chicago, IL, USA). Data were analyzed in two phases: during the pre-exercise and exercise periods. A two-way (drink × time) repeated-measures analysis of variance (ANOVA) was performed to compare the changes in the Tre, Tsk, HR, RPE, and RTS between the experimental conditions. The time to exhaustion (TTE), BM, USG, HS, and TSL physiological variables at exhaustion between the three experimental conditions were examined using a one-way (drink) repeated-measures analysis of variance ANOVA. When a significant main effect or interaction effect was identified, the differences were delineated using a Bonferroni adjustment. For all comparisons, significance was set at a *p* value <0.05. All figures are represented as the means ± SEM for clarity of presentation, and all other data are presented as the mean ± SD.

## Results

The volume of beverage consumed during the pre-exercise period was 530 ± 101 g for all treatments. The hydration state before and after the experiment is summarized in Table 1. The mean BM in the LRI, SRI, and CON trials were significantly decreased after the experiment (*p* < 0.05), but no significant differences were found among the different trials. There were no significant differences in USG and TSL among the different trials.

**Table 1:** The hydration state before and after experiment

	COOL		SRI		LRI	
	Before	After	Before	After	Before	After
Body mass (kg)	70.9 ± 14.0	70.5 ± 14.2*	70.7 ± 13.0	70.0 ± 13.2*	70.7 ± 13.4	70.0 ± 13.2*
Total sweat volume (mL)	0.39 ± 0.32		0.771 ± 0.60		0.629 ± 0.46	
Urine specific gravity	1.019 ± 0.003	1.022 ± 0.004	1.013 ± 0.010	1.019 ± 0.006	1.016 ± 0.006	1.018 ± 0.008

*Before vs. after (*p* < 0.05)

### Cycling TTE

**Fig. 2:**
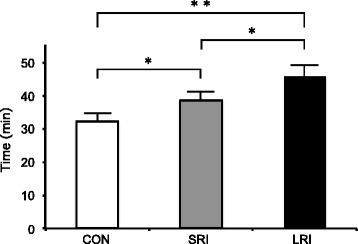
The cycling time to exhaustion under the three experimental conditions. *p < 0.05 and **p < 0.01

All participants cycled for a longer time in the LRI trial than in the SRI (*p* = 0.012) or CON trials (*p* = 0.001), and in the SRI trial than in the CON trial (Fig. 2; *p* = 0.01). The mean cycling time was 46.0 ± 7.7 min for the LRI trial, 38.7 ± 5.7 min for the SRI trial, and 32.3 ± 3.2 min for the CON trial, respectively.

### Tre and Tsk

**Fig. 3:**
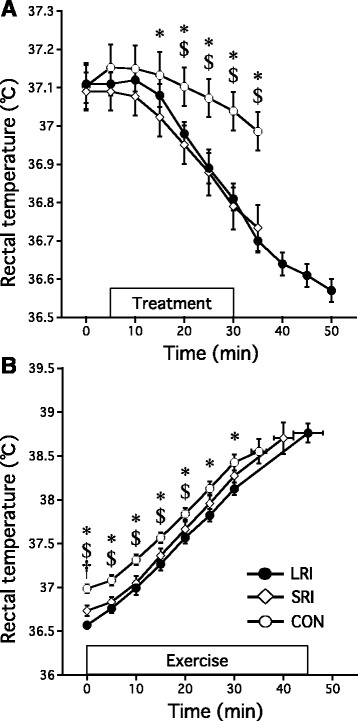
The rectal temperature before exercise (**a**) and during exercise (**b**) under the three experimental conditions. The mean values are expressed as mean ± SEM of seven participants (CON:○, SRI:◇, LRI:●). Time × drink effect CON vs. LRI: *p < 0.05, CON vs. SRI: ^$^ *p* < 0.05, and SRI vs. LRI: ^†^ *p* < 0.05

At rest, Tre was similar among trials (Fig. 3a). A LRI reduced Tre by 0.55 ± 0.07 °C (*p* = 0.001) immediately before the start of exercise and were significantly greater than in the SRI (0.36 ± 0.16 °C; *p* = 0.04) and CON trials (0.11 ± 0.14 °C; *p* = 0.001). The Tre increased progressively in each trial during exercise, but LRI or SRI trials reminded lower than the CON trial for the first 30 min of exercise (*p* = 0.001; Fig. 3b). The rate of rise in the Tre during exercise was not significantly different between trials (*p* > 0.05). Similarly, at exhaustion, Tre was not significantly different between trials, respectively (CON 38.56 ± 0.37 °C, SRI 38.70 ± 0.48 °C, LRI 38.76 ± 0.30 °C, *p* > 0.05). There were no significant differences in Tsk among the different conditions throughout trials throughout the study (*p* > 0.05).

### HS and HR

**Fig. 4:**
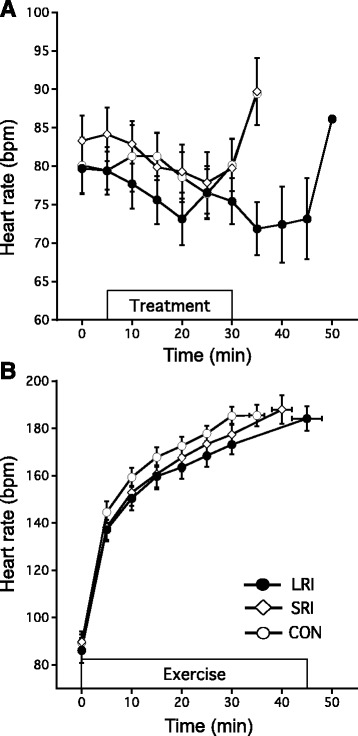
The heart rate before exercise (**a**) and during exercise (**b**) under the three experimental conditions. The mean values are expressed as mean ± SEM of seven participants (CON:○, SRI: ◇, LRI:●)

The HS in the LRI trial (−9.03 ± 4.61 W m^−2^) during the pre-exercise period was lower than that in the SRI (−4.98 ± 2.50 W m^−2^, *p* = 0.01) and CON trials (2.86 ± 4.44 W m^−2^, *p* = 0.001). During exercise, participants stored significantly more heat following a LRI (82.45 ± 11.52 W m^−2^) and SRI (77.58 ± 13.28 W m^−2^) than with CON (62.53 ± 15.41 W m^−2^, *p* < 0.05). However, the amount of heat stored was not different between the LRI and SRI trials (*p* = 0.734). There were no significant differences in HR among the different trials throughout the study (*p* > 0.05; Fig. 4).

### RTS and RPE

**Fig. 5:**
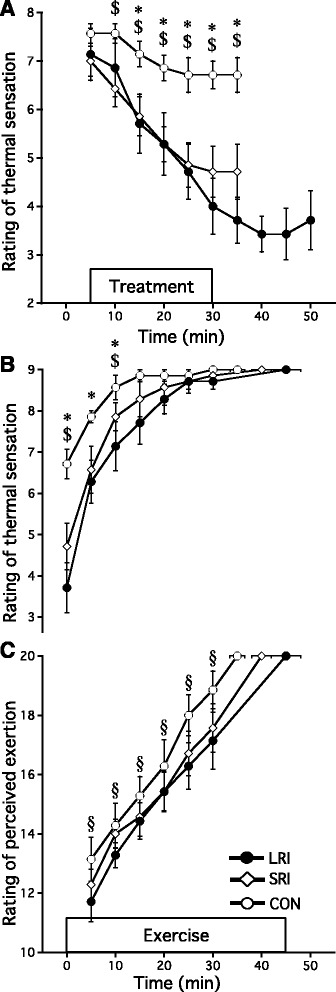
The rating of thermal sensation before exercise (**a**), during exercise (**b**), and rating of perceived exertion (**c**) under the three experimental conditions. The mean values are expressed as mean ± SEM of seven participants (CON:○, SRI:◇, LRI:●). Time × drink effect CON vs. LRI: *p < 0.05, CON vs. SRI: ^$^ *p* < 0.05, and CON vs. LRI: ^§^ *p* < 0.10

Measurements of the RTS and RPE are presented in Fig. 5. There were no significant differences in the RTS between trials immediately before the start of each treatment (*p* > 0.05). In the pre-exercise period, the RTS in the LRI trial decreased significantly from 15 to 35 min compared with the CON trial (*p* < 0.05), and the RTS in the SRI trial decreased significantly from 10 to 35 min compared with CON (*p* < 0.05). The RTS was significantly lower after ice ingestion than after cold water ingestion until the first 10 min of exercise (*p* < 0.05). The RPE in the LRI trial tended to be lower than in the CON from 0 to 30 min (*p* < 0.10). The RTS and RPE at exhaustion were similar between conditions (*p* = 1.000).

In the pre-exercise period, five of the seven participants experienced headaches while consuming ice, whereas none experienced this symptom with cold water ingestion. No participants reported any headaches or gastrointestinal discomfort during each trial when exercising.

## Discussion

The aims of this study were to compare the TTE and core temperature from ice ingestion with long rest (LRI, novel strategy) and short rest strategy (SRI, traditional strategy) before and during cycling capacity tests in the heat. In accordance with our hypothesis, ice ingestion was significantly more effective in extending the cycling TTE than cold fluid ingestion (CON 32.3 ± 3.2 min) and the TTE was extended further with a long rest interval (LRI 46.0 ± 7.7 min) than a short rest interval (SRI 38.7 ± 5.7 min).

The results of the present study showed that Tre in the LRI trial continued to drop until 20 min after ingestion and resulted in a greater reduction in Tre (−0.55 ± 0.07 °C) than in the SRI (−0.36 ± 0.16 °C) and CON trials (−0.11 ± 0.14 °C). In addition, the HS in the LRI trial during the pre-exercise period was lower than that observed in the SRI (−4.98 ± 2.50 W m^−2^) and CON trials (2.86 ± 4.44 W m^−2^). Previous studies have noted that the timing of ice ingestion when individuals are performing internal pre-cooling may be important for enhancing endurance performance and reducing the core temperature [16, 17]. Several previous studies investigated various timings in pre-cooling with ice ingestion. Siegel et al. [9] reported that the ice ingestion of 1.25 g kgBM^−1^ every 5 min in the 30 min prior to exercise, including 5-min rest after the end of ingestion, was able to significantly reduce the Tre by 0.66 °C and improved the running TTE at the first ventilatory threshold (VT) by 19% compared with cold water ingestion. In a separate study, the same authors [17] found that ice ingestion of 1.25 g kgBM^−1^ every 5 min in the 40 min prior to exercise continued to reduce the Tre during 10 min of rest afterwards, (from immediately after ice ingestion until just before the start of exercise) and improved the running TTE at first VT by 13% compared with warm water ingestion. However, measurements such as thermoregulatory responses and exercise capacity were not assessed due to different conditions in previous studies. In the previous study [15], Tre at approximately 20 min after the end of ice ingestion of 1.25 g kgBM^−1^ every 5 min tended to be lower than at 5 min after ingestion. Therefore, the ingestion of ice with a LRI resulted in a greater reduction in Tre and enhanced the heat sink effects before exercise as well as improved the endurance cycling capacity compared with the ingestion of ice with an SRI.

In addition to these effects, ice ingestion with a long rest interval may also reduce the brain temperature. If ice ingestion attenuates central fatigue, a reduction in the brain temperature might contribute to improved exercise performance [24]. Internal cooling by the ingestion of ice prior to exercise may result in the conductive cooling of the brain because ice is ingested through the mouth [17]. Ice ingestion may cool the blood flowing to the brain, thereby reducing the brain temperature during exercise as similar to the effect of neck cooling [25]. Onitsuka et al. [14] also reported that the ingestion of ice continued to decrease the forehead skin temperature until approximately 10 min after the end of ingestion. These authors concluded that ice ingestion may reduce brain temperature by conductive cooling from the facial area. Thus, the ingestion of ice with long rest might result in a lower brain temperature before exercise than that with short rest, an effect that might contribute to improved endurance exercise capacity.

The attainment of a critical limiting temperature of approximately 40 °C has been proposed as one of the main factors limiting endurance performance in hot environments [4]. It is suggested that this critical limiting temperature is used as a set point, around which subjects experienced exercise exhaustion. The core temperature at exhaustion in the present study was lower than the previously referenced critical level of 40 °C. In contrast, Cheung and McLellan [5] compared TTE between highly and moderately trained subjects and found that the highly trained group reached exhaustion at ~39.2 °C in Tre, while the moderately trained group reached exhaustion at ~38.8 °C in Tre, suggesting that training status influenced hyperthermia tolerance. Furthermore, Gagnon et al. [26] showed that Tre at the end of exercise was significantly lower than esophageal temperature in a hot environment. These findings therefore suggested that a critical limiting temperature existed in the present study and which might differ depending on the level of aerobic fitness, motivation for hyperthermia tolerance, or measurement sites of the body temperature. However, a recent review proposed a complex and integrated model of the mechanisms associated with exercise heat stress, including perturbations in the body temperature, aerobic fitness, and oxygenation [3, 27]. Further studies are therefore need ed to examine the effects of such factors and performance improvements in a hot environment.

Reductions in the RTS toward feeling less hot may be important for endurance exercise in hot environments. In the present study, ice ingestion (LRI and SRI trials) reduced the RTS before and during exercise compared with cold water ingestion (CON trial), which is in agreement with the finding of previous studies [9]. One potential factor that might influence the reduction of the RTS by ice ingestion is a change in the afferent feedback signals from the gastrointestinal tract. Sensory stimulation may be greater with ingestion due to extensive contact with thermoreceptors in the gastrointestinal tract [28]. The combination of extensive sensory stimulation and cooling of the gastrointestinal tract elicited a greater reduction in thermal sensation [29]. In addition, McArthur and Feldman [30] reported that the instillation of 360 mL cold meal (4 °C) induced the maximum change in the intragastric temperature 2.8 min after beginning instillation, after which the intragastric temperature rapidly increased. This theory is supported by the present data of no significant differences in the RTS between rest times. Ingestion of 10 g kgBM^−1^ of ice slurry during exercise also resulted in a reduction in the intragastric temperature, which rose after ingestion [31]. Thus, there are probably thermoreceptors in the stomach regions, which sense cold sensation by ice ingestion and transmit afferent feedback signals regarding the intragastric temperature, thereby reducing the RTS.

The present study is associated with some limitations. First, this study followed a TTE protocol. We used this protocol to assess the attainment of a critical core temperature. Further studies are therefore needed to examine the results of performance tests that are more ecologically valid such as discrete tests set for time or distance. Second, the sample used in the present study is not representative of participants who would potentially undertake prolonged exercise during hyperthermic conditions. Finally, the participants in the present study were not trained. A previous study found that the Tre in trained participants at exhaustion was higher than that in untrained participants [5]. Nielsen et al. [32] suggested that a highly fit group would be longer motivated to exercise and have the ability to reach a greater core temperature than less fit subjects. Additionally, human-related factors such as motivation may play a role in the variation seen in hyperthermia tolerance among individuals [33].

## Conclusion

In conclusion, our results indicate that ice ingestion with a long rest interval reduced the Tre and HS before exercise, thereby enhancing the exercise capacity in hot environments as compared with that following a short rest or with cold water ingestion. The present results suggested that ice ingestion with long rest before exercise may be more ergogenic than previously published strategies.

## Abbreviations

BM: Body mass
HR: Heart rate
HS: Heat storage
LRI: Long rest interval
$\overset{.}{\mathrm{V}}$ O_2_max: Maximal oxygen uptake
RH: Relative humidity
RPE: Rating of perceived exertion
RTS: Rating of thermal sensation
SRI: Short rest interval
Tb: Mean body temperature
Tre: Rectal temperature
Tsk: Mean skin temperature
TTE: Time to exhaustion
USG: Urine specific gravity
VT: Ventilator threshold
